# CRISPR-based assays for the detection of BK virus and JC virus infections post-kidney transplantation

**DOI:** 10.1186/s40779-025-00632-0

**Published:** 2025-08-04

**Authors:** Yu Liu, Jing-Song Xu, Li Cao, Shuang Yang, Tian-Ming Li, Hai-Qian Huang, Jun-Heng Zhang, Xue Zhao, Qian Liu, Shun Li, Min Li, Hua Wang

**Affiliations:** 1 Department of Laboratory Medicine, Renji Hospital, School of Medicine, Shanghai Jiao Tong University, Shanghai, 200127 China; 2 Department of Pathology, the Fifth People’s Hospital of Shanghai, Fudan University, Shanghai, 200240 China; 3 Central Laboratory, Renji Hospital, School of Medicine, Shanghai Jiao Tong University, Shanghai, 200127 China; 4 Shanghai Public Health Clinical Center, Fudan University, Shanghai, 201508 China

**Keywords:** BK virus (BKV), JC virus (JCV), Kidney transplantation, Clustered regularly interspaced short palindromic repeats (CRISPR), Microfluidic, Lateral flow assay (LFA)

## Abstract

**Background:**

Organ transplantation recipients encounter significant risks from acute or chronic infections that threaten graft survival. BK virus (BKV) and JC virus (JCV) are two prominent opportunistic infection viruses, and they may cause polyomavirus-associated nephropathy and graft kidney loss in patients who are in an immunosuppressed state after kidney transplantation. Hence, timely detection and sustained monitoring of the viral load are indispensable. However, the current diagnostic methods remain limited, and the development of new molecular detection technology is extremely urgent.

**Methods:**

The sequences and concentrations of clustered regularly interspaced short palindromic repeats (CRISPR) RNA (crRNA), the concentration of Cas13a, and the primers for recombinase polymerase amplification (RPA) were optimized for BKV and JCV detection. Next, a novel microfluidic dual-droplet chip was designed and fabricated, and it was integrated with CRISPR (ddCRISPR) to simultaneously qualitatively detect BKV and JCV. Subsequently, the ddCRISPR assay was verified using clinical samples. Then, a lateral flow strip combined with CRISPR (LFCRISPR) was developed for the detection of BKV and JCV in resource-limited settings.

**Results:**

A one-pot RPA-CRISPR reaction system was established and optimized for BKV and JCV detection. ddCRISPR can simultaneously and rapidly detect BKV and JCV with high sensitivity (10 copies/ml for BKV and 1 copy/ml for JCV), and provide absolute quantification, which is suitable for viral load detection and conducive to personalized and precise treatment for organ transplant recipients. LFCRISPR simplified the operational process through a simple visual readout, facilitating virus screening after organ transplantation.

**Conclusions:**

These platforms incorporate molecular testing into the transplantation treatment model, thereby reducing costs, prolonging the survival time of the graft, improving the clinical outcomes of postoperative management in kidney transplantation, and enhancing the patients’ quality of life.

**Supplementary Information:**

The online version contains supplementary material available at 10.1186/s40779-025-00632-0.

## Background

Polyomaviruses (PyVs), a class of circular double-stranded DNA (dsDNA) viruses, are known to potentially cause opportunistic infections in immunocompromised individuals [1]. Among PyVs, the BK virus (BKV) and JC virus (JCV) are two prominent types that are prevalent in the global population but typically remain suppressed under normal immune conditions [2]. Primary infections with BKV or JCV generally occur in childhood or adolescence, but it is often asymptomatic in healthy individuals and then enter a latent period [3]. However, there is a high risk of BKV activation among immunocompromised populations, leading to local urinary system infections such as hemorrhagic cystitis, nephritis, and urinary tract infections [4, 5]. Additionally, infection with JCV in immunocompromised individuals can result in progressive multifocal leukoencephalopathy (PML), manifesting as a series of progressive psychiatric symptoms, altered consciousness, and even irreversible damage to the central nervous system [6–8]. Increasing evidence suggested that infections with BKV and JCV are also associated with polyomavirus-associated nephropathy (PVAN) in patients undergoing immunosuppressive therapy following renal transplantation, which may lead to impaired kidney function or graft loss [9–11]. Furthermore, several studies have demonstrated that delays in reporting BKV or JCV infections among these populations can lead to adverse clinical outcomes, such as persistent infection or increased mortality [12, 13]. Therefore, the development of rapid and highly accurate technologies for BKV/JCV detection is imperative to reduce the risks associated with delayed or false-negative diagnoses. Such advancements would enable timely adjustment of immunosuppressive regimens and proactive infection monitoring, ultimately lowering mortality rates among kidney transplant recipients.

To date, a variety of diagnostic methods have been extensively utilized for the detection of BKV and JCV, including histological biopsy, urinary cytology, and quantitative polymerase chain reaction (qPCR) [14]. Histological biopsy, regarded as the gold standard for diagnosing PVAN, enables experienced pathologists to deliver precise diagnostic information and stage the disease. However, as an invasive procedure, it is limited by sampling error, challenging in distinguishing between BKV and JCV infections, and poses a number of significant risks for patients, such as bleeding and high costs [15]. Although urinary cytology is non-invasive and convenient for urine sample collection, it is also prone to subjective interpretation by reporters and may be unable to confirm infection when no decoy cells (a hallmark of BKV infection) are identified [11, 14]. As the molecular diagnostic gold standard, qPCR has been extensively utilized for viral load quantification [16–18]. Nonetheless, the requirement for a PCR laboratory, substantial labor, and specialized equipment restricts its applicability as a point-of-care test in resource-limited settings. Recombinase polymerase amplification (RPA) is a nucleic acid detection technology that is referred to as an alternative to PCR, with the advantages of high sensitivity, compatibility with constant-temperature reaction, and no need for expensive equipment [19]. Due to its outstanding performance, RPA has garnered increasing attention [20–22]. However, the non-specific amplification during the RPA assay can lead to false-positive results [23]. Recently, the clustered regularly interspaced short palindromic repeats (CRISPR)/Cas system has emerged as a promising nucleic acid detection technology [24–27]. Activated by CRISPR RNA (crRNA) binding to its specific RPA products, the Cas endonuclease generates fluorescence signals by trans-cleavage of fluorescence reporters. This process effectively compensates for RPA’s non-specific amplification and prevents the occurrence of false-positive results. The RPA-CRISPR detection system demonstrated robust, sensitive, and specific characteristics in molecular diagnostics [28–30]. We previously reported a strategy for the rapid and sensitive detection of JCV by integrating a microfluidic device into the RPA-CRISPR system [31]. Nevertheless, this method has certain limitations. The chip proposed in this work lacks integration. After droplet formation, the droplets were collected in a tube for the RPA-CRISPR reaction and then transferred to a detection chip for analysis. Furthermore, clinical observations indicated that patients receiving kidney transplantation often present with co-infections of BKV and JCV, necessitating simultaneous detection and ongoing monitoring of these viruses.

To address the aforementioned challenges, this study aims to develop two innovative and efficient diagnostic platforms. These platforms integrate RPA-CRISPR-based detection with microfluidic technology and are specifically designed for the monitoring and screening of BKV and JCV. The objective is to establish a scientifically rigorous basis for clinical diagnosis and therapeutic decision-making, thereby facilitating improved patient outcomes.

## Methods

### Materials

The RNase inhibitor (Cat No. B300076), T7 transcriptase (Cat No. B110085), and nucleoside triphosphate (NTP) mixture (Cat No. B600056) were purchased from Sangon Biotech Co. Ltd., Shanghai, China. The BKV (Cat No. 20163402071) and JCV (Cat No. 20163402078) nucleic acid quantitative PCR detection kits were purchased from Beijing Sinomdgene Technology Co., Ltd., Beijing, China. The RPA amplification kits (Cat No. DNA-LS01) and LFA strips (Cat No. TEC-LS05) were purchased from LeSun Biotechnology Co. Ltd., Wuxi, China. Cas13a protein (Cat No. C005S/M) was purchased from Magigen Bio, Guangzhou, China. The poly(dimethylsiloxane) (PDMS) and curing agent (Cat No. H052N7A066), SU-8 3050 (Cat No. SP03546), and SU-8 2075 (Cat No. SP01816) were purchased from Suzhou Cchip Scientific Instrument Co., Ltd., Suzhou, China. All primers, crRNAs, single-strand RNA (ssRNA) reporters, BKV, JCV, Merkel cell polyomavirus (MCPyV), Washington University polyomavirus (WUPyV), Karolinska Institute polyomavirus (KIPyV), human polyomavirus 6 (HPyV6), and human polyomavirus 7 (HPyV7) plasmid standards were synthesized by Sangon Biotech Co., Ltd., Shanghai, China. The sequences of all primers, crRNAs, and reporters are listed in Additional file 1: Table S1.

### Methodology overview

**Fig. 1:**
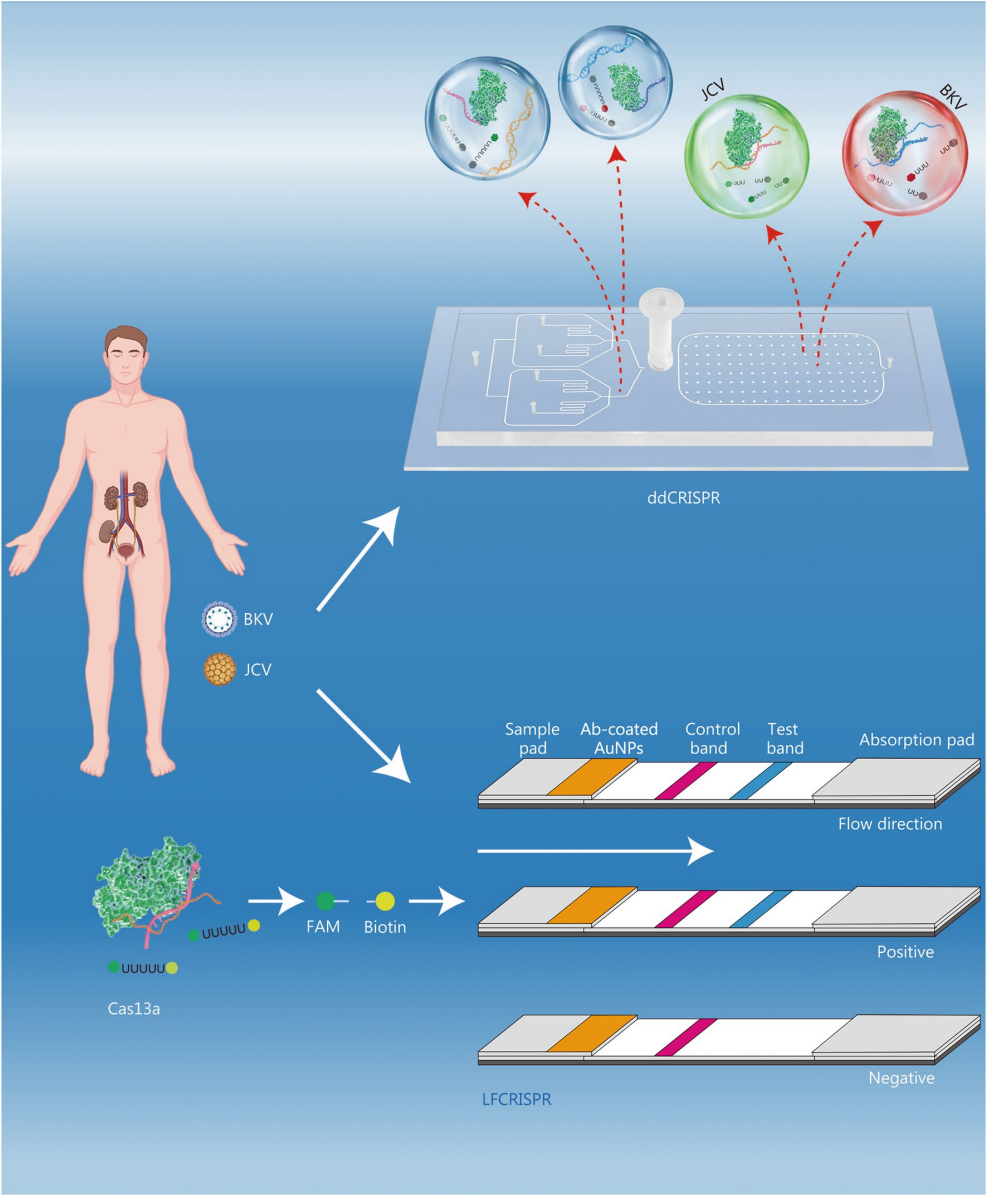
Schematic diagram of ddCRISPR and LFCRISPR for BK virus (BKV) and JC virus (JCV) detection in a patient’s post-kidney transplantation. Following renal transplantation, recipients infected with BKV and JCV were detected or continuously monitored using ddCRISPR, while LFCRISPR was applied for early infection screening. ddCRISPR dual-droplet microchip integrated with RPA-CRISPR, LFCRISPR lateral flow strip integrated with RPA-CRISPR, FAM 6-carboxyfluorescein, AuNPs gold nanoparticles

In this study, a self-fabricated dual-droplet microchip and lateral flow strip were integrated into RPA-CRISPR to develop detection of BKV and JCV, referred to as ddCRISPR and LFCRISPR, respectively (Fig. 1). By combining with an integrated dual-droplet microchip, ddCRISPR can simultaneously detect two targets using distinct fluorescent probes and enable simultaneous absolute quantification based on digital droplets. To fulfill the clinical demand for the rapid screening of BKV and JCV, we have developed a CRISPR-based lateral flow (LFCRISPR) assay for point-of-care testing of BKV and JCV.

### Dual-droplet microchip design and fabrication

The construction of the dual-droplet microchip was designed using AutoCAD 2022 (Autodesk, lnc., San Rafael, CA, USA). To fabricate the mold for the microchip, we used photolithography technology. The pre-cleaned silicon wafer was spin-coated with SU-8 3050 (500 rpm, 10 s; 4000 rpm, 30 s). Then, the wafer was baked on a hot plate at 95 ℃ for 15 min. Next, an ultraviolet exposure (200 mJ/cm^2^) was conducted to transfer the graphics to the wafer. A post-exposure bake (65 ℃, 1 min; 95 ℃, 5 min) was followed. Subsequently, the wafer was coated with SU-8 2075 (500 rpm, 10 s; 2000 rpm, 30 s) and baked (65 ℃, 5 min; 95 ℃, 20 min). Afterwards, it was photolithographed by ultraviolet (215–240 mJ/cm^2^). Finally, the wafer was treated using a developer.

For chip fabrication, a mixture of 10:1 (w/w) PDMS and curing agent was poured into the mold. After heating at 85 ℃ for 2 h, the chip was cut off and then performed with punching and plasma-treatment. Next, the microchannels of the chip were hydrophobised using a hydrophobic agent and then reheated at 85 ℃ for 10 min. In the end, a leak-proof and hydrophobic dual-droplet microchip was ready.

### One-pot RPA-CRISPR assay

In this procedure, the RPA freeze-dried pellet was first dissolved by adding 25 μl RPA buffer. Then, the premix solution was prepared, which contained 5 μl NTP, 2 μl crRNA, 1 μl forward primer, 1 μl reverse primer, 1 μl DNA template, 2 μl ssRNA reporter, 4 μl Cas13a, 1 μl RNase inhibitor, 0.6 μl T7 transcriptase, and 5.4 μl buffer. Next, the solution was transferred to the tube containing the RPA reagent. After being initiated by adding 2 μl of initiator, the reaction assay was performed at 37 ℃ for 30 min, and the fluorescence signal was monitored by using a real-time PCR system or microplate reader.

### ddCRISPR assay

The droplet assay was made into two separate mixes for loading onto the microfluidic chip. The components of mix 1 to detect BKV or JCV were different, which contained an RPA freeze-dried pellet dissolved by 11 μl RPA buffer, 5 μl NTP, 2 μl BKV or JCV’s crRNA, 1 μl forward primer, 1 μl reverse primer, 2 μl template, and 4 μl ssRNA reporter [cyanine5 (CY5) for BKV or 6-carboxyfluorescein (FAM) for JCV]. The components of mix 2 to detect BKV or JCV were the same, which contained 4 μl Cas13a, 1 μl RNase inhibitor, 0.6 μl T7 transcriptase, 17.5 μl RPA buffer, and 2 μl initiator. After preparing the mixture and loading it into the device, a droplet generator aided by a pressure pump was used to drive and control the droplet generation. The droplets were produced by the flow-focusing junction and subsequently incubated in the detection zone at 37 ℃ for 30 min, followed by imaging the droplets and analyzing the results.

### Optical imaging system and image processing

The droplets were imaged and characterized by an inverted fluorescent microscope Nikon Ti2-U, which enabled both bright-field images and fluorescent images to be captured. The obtained pictures were further analyzed by the ImageJ software (National Institutes of Health, USA). The steps were processed as: “Image” “Color” “Channels Tool” and “Choosing Red/Green channel”. And the merged image was processed as follows: “Image” “Color” “Merge Channels” and “Choosing Red and Green channels”.

### Digital quantification

According to the volume of chip storage area, up to 40,000 droplets were generated and then analyzed after incubation in each experiment. The target concentration (*C*) was obtained according to the Poisson distribution, which can be calculated by:

$$ C = - \frac{{\ln \left( {1 - PPD} \right)}}{V},\;V = \frac{1}{6}\pi D^{3} $$

where *PPD* is the proportion of positive droplets, and droplet volume (*V*) is estimated by the diameter of droplets (*D*). The fluorescence intensity of the droplet was analyzed by the Nebula auto digital PCR system (ThunderBio Life Sciences, Jiaxing, China), a chip reading device.

### LFCRISPR assay

The quenchers modified on reporters are replaced by biotin molecules [FAM-poly (U)-Biotin]. The general reaction system (25 μl) is essentially the same as the one-pot RPA-CRISPR reaction (shown in the One-pot RPA-CRISPR assay section). The reaction was carried out at 37 ℃ for 30 min and then mixed with 25 μl nuclease-free water. After mixing, the LFA strip was immersed in the solution and incubated for 5 min. Then, the strips were taken out, and images were captured using a smartphone. The LFA images were analyzed using ImageJ software (National Institutes of Health, USA).

Detailed information of other methods is provided in Additional file 1: Methods.

### Statistical analysis

Unless otherwise specified, technical replicates were performed in triplicate, and all illustrations were drawn by GraphPad Prism version 9.5.1 (GraphPad Software, San Diego, CA, USA) and processed by Adobe Illustrator 2021 (Adobe Inc., San Jose, CA, USA). Mean ± standard deviation (SD) was calculated with standard formulas in Excel. The McNemar test was performed to compare the sensitivity and specificity of ddCRISPR and qPCR. To evaluate the correlation between qPCR Ct values and the logarithmic values of ddCRISPR-quantified copy numbers, simple linear regression was performed using GraphPad Prism software. Statistical differences of LFCRISPR detection results between the control group and infected group were conducted using a two-tailed Student’s *t*-test. *P* < 0.05 was considered statistically significant.

## Results

### Establishment and optimization of one-pot RPA-CRISPR assay

**Fig. 2:**
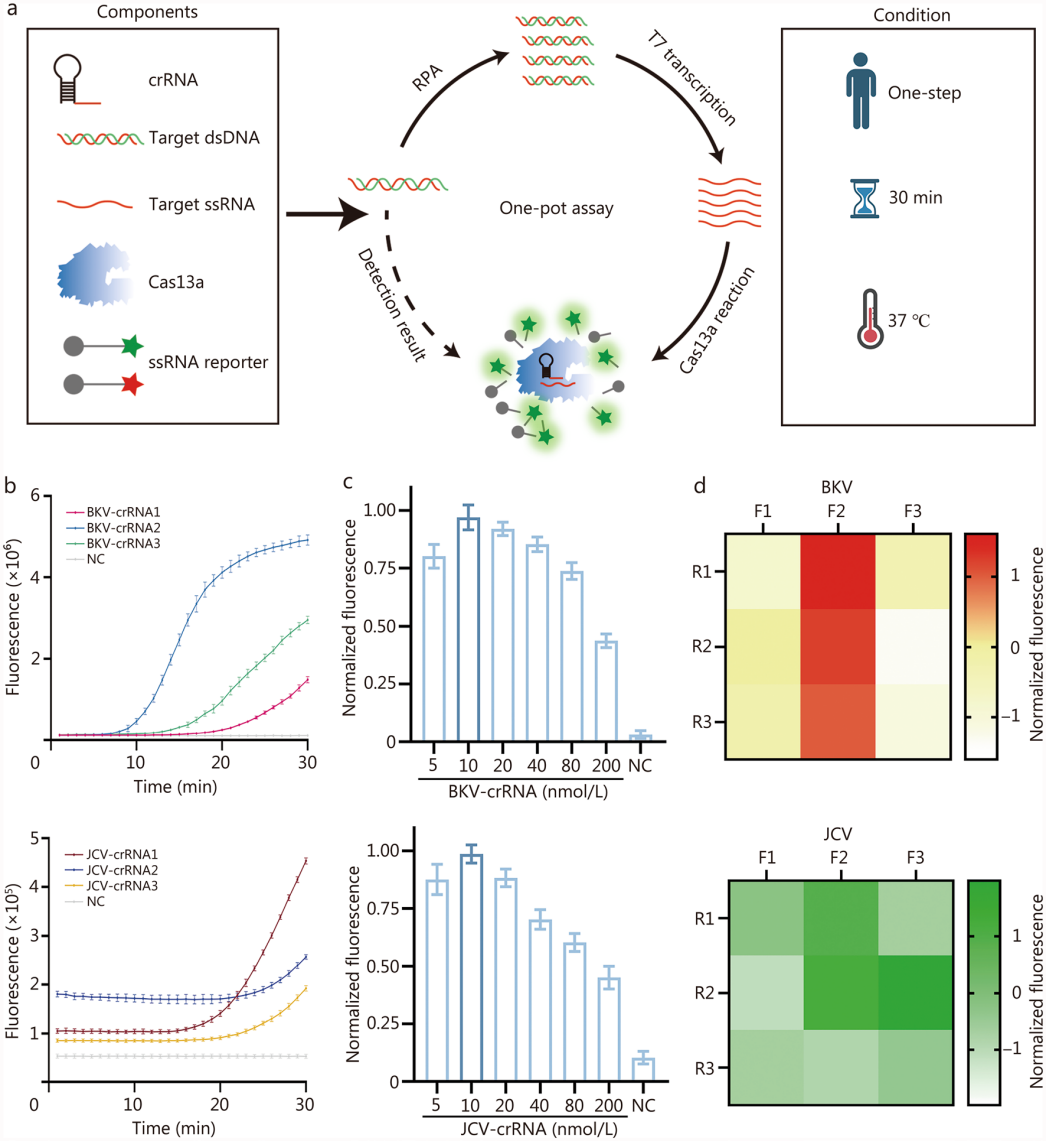
Establishment and optimization of one-pot RPA-CRISPR assay. **a** Schematic illustration of one-pot RPA-CRISPR assay. Target DNA is amplified using RPA. Subsequently, RNA transcription is performed using T7 RNA polymerase. Finally, the trans-cleavage activity of Cas13a is activated upon the binding of crRNA to target RNA, facilitating fluorescence detection. **b** Comparison of BKV and JCV detection efficiency using three different crRNAs (*n* = 3). **c** Optimization of the concentration of BKV-crRNA and JCV-crRNA (*n* = 3). **d** Heatmap of BKV and JCV primer optimization (*n* = 3). The results were normalized (**b**, **c**, **d**). Data are presented as mean ± standard deviation. crRNA clustered regularly interspaced short palindromic repeats RNA, dsDNA double-stranded DNA, ssRNA single-strand RNA, RPA recombinase polymerase amplification, BKV BK virus, JCV JC virus, NC negative control

The assay was initiated by introducing samples to an all-in-one mixture containing the components of RPA, RNA transcription, and CRISPR reaction. The entire process was accomplished at 37 ℃ within 30 min. As shown in Fig. 2a, RPA was adopted to generate the amplicons of BKV and JCV, and the T7 promoter sequence was added to the primers to achieve T7 RNA polymerase-mediated transcription. After the crRNA binds to the RNA target with complementary sequences, it activates LwaCas13a to perform trans-cleavage on the ssRNA reporter molecule carrying the quenched fluorophore, thereby enabling fluorescence detection of the target dsDNA.

Based on the aforementioned experimental procedures, we initially optimized the one-pot RPA-CRISPR assay in tubes. Three crRNAs targeting distinct sequences of BKV or JCV were designed for the optimization of the crRNA sequence. As demonstrated in Fig. 2b, BKV-crRNA2 and JCV-crRNA1 were identified as the optimal conditions for subsequent experiments. Next, a series of crRNA dilutions (5 to 200 nmol/L) were evaluated to determine the optimal concentration. As illustrated in Fig. 2c, the fluorogenic signals initially increased with increasing crRNA concentration, reaching a peak at 10 nmol/L, and then decreased at higher concentrations. No signal was observed in the absence of crRNA. Similar to crRNA, varying the concentration of Cas13a could also affect the fluorescence signal. To determine the optimal concentration of Cas13a, we conducted a comprehensive screening within a range of concentrations (20 to 400 nmol/L), and eventually identified 100 nmol/L as the optimal concentration (Additional file 1: Fig. S1).

In terms of RPA optimization, we designed 3 pairs of RPA primers for the detection of BKV and JCV, respectively. Figure 2d indicates that the primer combination BKV F2R1 and JCV F3R2 achieves the highest fluorescence signal, thus they are the optimized combinations. Furthermore, Sanger sequencing was performed to validate that RPA achieved effective amplification of BKV and JCV (Additional file 1: Fig. S2). These optimal conditions were selected for the subsequent study. Aside from BKV and JCV, we included several other human PyVs subtypes, including KIPyV, WUPyV, MCPyV, HPyV6, and HPyV7, and two other transplantation-associated viruses, cytomegalovirus (CMV) and Epstein-Barr virus (EBV), in the specificity assessment [32, 33]. The findings indicated an absence of cross-reactivity among the 7 viruses, thereby confirming the specificity of the assay (Additional file 1: Fig. S3).

### Evaluation of the ddCRISPR assay

**Fig. 3:**
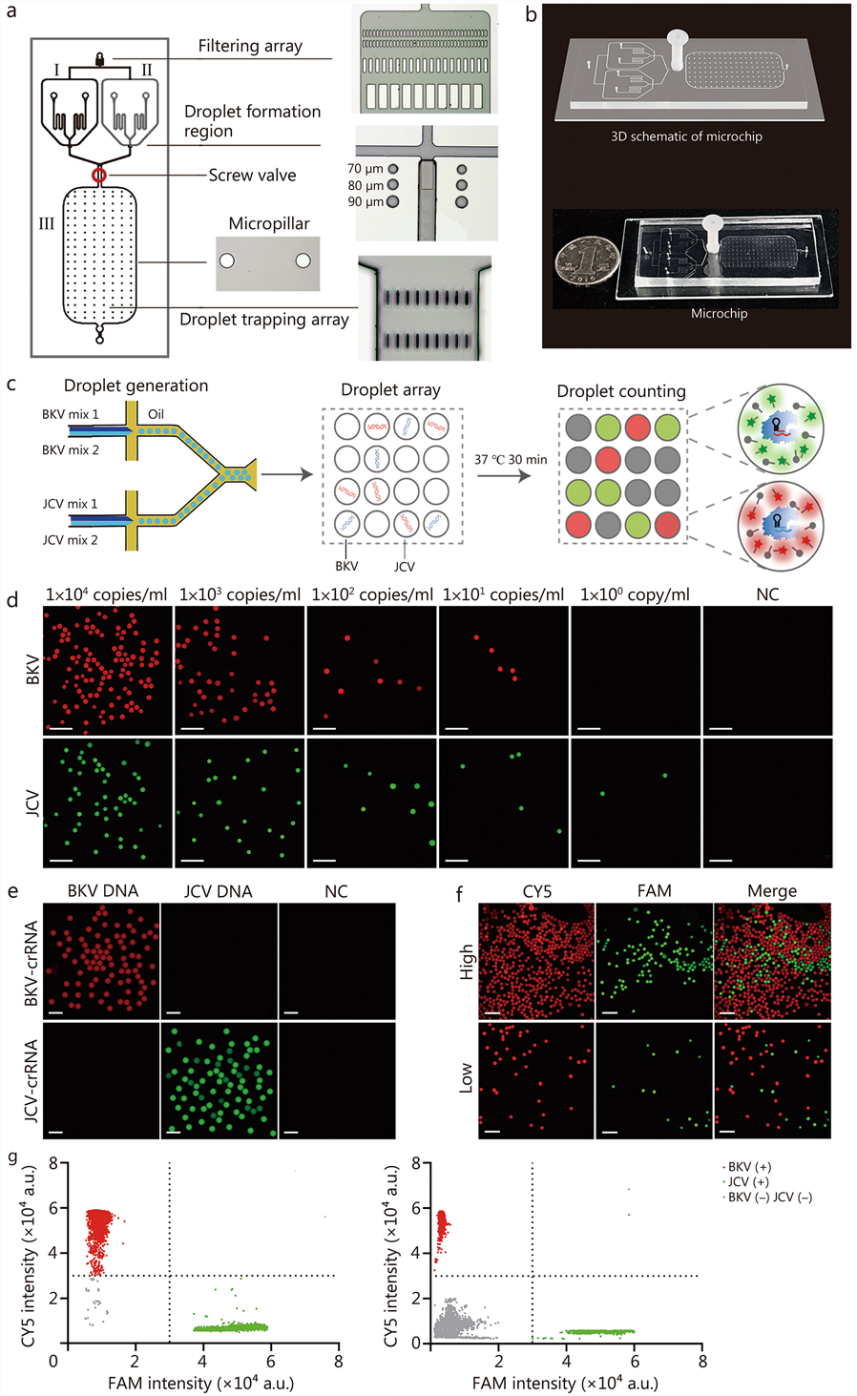
Evaluation of the ddCRISPR assay. **a** Basic structure of a dual-droplet microchip. **b** 3D illustration (top) and physical illustration (bottom) of a microchip. **c** Workflow of ddCRISPR assay. After two channels of droplet generation, all droplets are incubated at 37 ℃ for 30 min and detected in a fluorescent field for result readout. **d** Representative fluorescence images of a serial dilution of DNA concentrations of BKV and JCV detection for sensitivity evaluation. Scale bar = 500 μm. **e** Representative results of the specificity evaluation. Scale bar = 200 μm. **f** Representative fluorescence images showing the simultaneous detection capability using a mixture with high- and low-concentration. Left, droplets encapsulating CY5 for BKV detection; Middle, droplets encapsulating FAM for JCV detection; Right, merged image of left and middle images. Scale bar = 500 μm. **g** Scatter plots showing high- (left) and low-concentration (right) mixture, respectively. I and II indicate droplet generation unit, III indicates droplet detection zone, BKV BK virus, JCV JC virus, NC negative control, crRNA clustered regularly interspaced short palindromic repeats RNA, FAM 6-carboxyfluorescein, CY5 cyanine5, BKV (+) BK virus (positive), JCV (+) JC virus (positive), BKV (-) JCV (-) BK virus (negative) and JC virus (negative), ddCRISPR dual-droplet microchip integrated with RPA-CRISPR, 3D three-dimensional

As shown in Fig. 3a, the dual-droplet chip consists of two main parts, the droplet generation zone and the droplet detection zone. The chip design corresponds to the structure of the SU-8 mold. It has a droplet generation zone, which includes a BKV droplet generation unit (I) and a JCV droplet generation unit (II). The micropillars arranged in the droplet detection zone (III) can support the structure of the chip and prevent droplet collapse. Additionally, the oil phase inlet is equipped with a filter array, which is capable of filtering out impurities in the oil. The droplet trapping array is positioned at the liquid outlet to prevent droplets from flowing out of the outlet. The droplet interception array and screw valve confine the droplet within the detection zone for the accurate detection of BKV and JCV. The 3D schematic and physical images of the chip are presented in Fig. 3b. Considering the working principle of digital droplet analysis, the accuracy and reproducibility of ddCRISPR quantification required significant droplet uniformity in terms of size and number. Subsequently, experiments were performed. As observed under microscopy, the droplets generated by the dual-droplet microchip were highly consistent in size (Additional file 1: Fig. S4a). Particle analysis revealed an average diameter of (92.43 ± 4.41) μm, corresponding to a coefficient of variation (CV) of 4.77%, confirming the high uniformity (Additional file 1: Fig. S4b). Given that the internal dimensions of the chip are precisely determined and highly consistent, the number of droplets generated in each experiment is also highly consistent when droplet size uniformity is maintained. We further conducted droplet generation experiments, analyzing the particle size of all droplets within the chip (with an average of up to 40,000 droplets per test). As shown in Additional file 1: Fig. S4c, we utilized 10 chips to validate the consistency and reproducibility of the generated droplet size. Given that ddCRISPR is conducted in a controlled, clean environment, the primary environmental factor influencing droplet behavior during the heating process is temperature. We have also included comparative analyses of droplet size pre- and post-heating, which confirmed that the droplets retain their dimensional integrity during the thermal treatment process (Additional file 1: Fig. S4d).

The workflow of the ddCRISPR assay is illustrated in Fig. 3c. The entire analysis process primarily comprises droplet generation, droplet array formation for RPA-CRISPR reaction, and droplet counting for absolute quantification. The droplet formation process proceeds as follows. Pressure is applied to both the oil phase and liquid phase, generating water-in-oil droplets containing BKV and JCV at the intersection of droplet generation I and II (Additional file 2: Video S1). These droplets are subsequently collected into the droplet storage and detection area III of the chip under pressure (Additional file 3: Video S2). Once the droplet storage area III is filled with droplets, the pressure is turned off, the chip is sealed, and the subsequent incubation reaction proceeds. Next, quantitative droplet detection is performed. To enable the simultaneous detection of two viruses within the chip, droplets for BKV detection contained CY5 probes, and droplets for JCV detection contained FAM probes. Finally, the quantitative results were obtained by counting the positive droplets using a chip reading device based on the Poisson distribution. All results detected by ddCRISPR (copies/ml) referred to the viral loads in the serum or urine.

This study assessed the sensitivity and specificity of on-chip assays for the detection of BKV and JCV under optimized conditions. The sensitivities of the assay were evaluated by testing serially diluted BKV and JCV standards. As illustrated in Fig. 3d, the findings indicated that the assay can detect as low as 10 copies/ml of BKV and JCV at single-molecule detection levels. In contrast, the detection limit of qPCR for both BKV and JCV was 100 copies/ml (Additional file 1: Fig. S5). Thus, this assay demonstrated greater competitiveness than qPCR in detecting low-concentration samples. Subsequent experiments confirmed no cross-reactivity between BKV/JCV reaction systems and PyVs or organ transplantation-related viruses from the same genus (Fig. 3e; Additional file 1: Fig. S6). To validate the capacity of simultaneous and quantitative detection of dual targets, a high-concentration mixture of BKV (10^6^ copies/ml) and JCV (10^5^ copies/ml) standards and a low-concentration mixture standard (BKV was at 10^3^ copies/ml and JCV at 10^2^ copies/ml) were utilized, respectively. As shown in Fig. 3f, both high- and low-concentration mixtures were accurately detected via ddCRISPR. The corresponding fluorescence scatter plots were obtained through the chip reading device [Fig. 3g (left) for high-concentration and Fig. 3g (right) for low-concentration].

In summary, we successfully established a ddCRISPR assay for the simultaneous detection of BKV and JCV. This assay was characterized by its higher sensitivity (10 copies/ml for BKV and 1 copy/ml for JCV) compared to qPCR (100 copies/ml for both BKV and JCV), excellent specificity, the ability to simultaneously detect two targets, and a reduced detection turnaround time (5 min for droplet generation, 30 min for incubation, and 2 min for result readout).

### The clinical application of the ddCRISPR assay

**Fig. 4:**
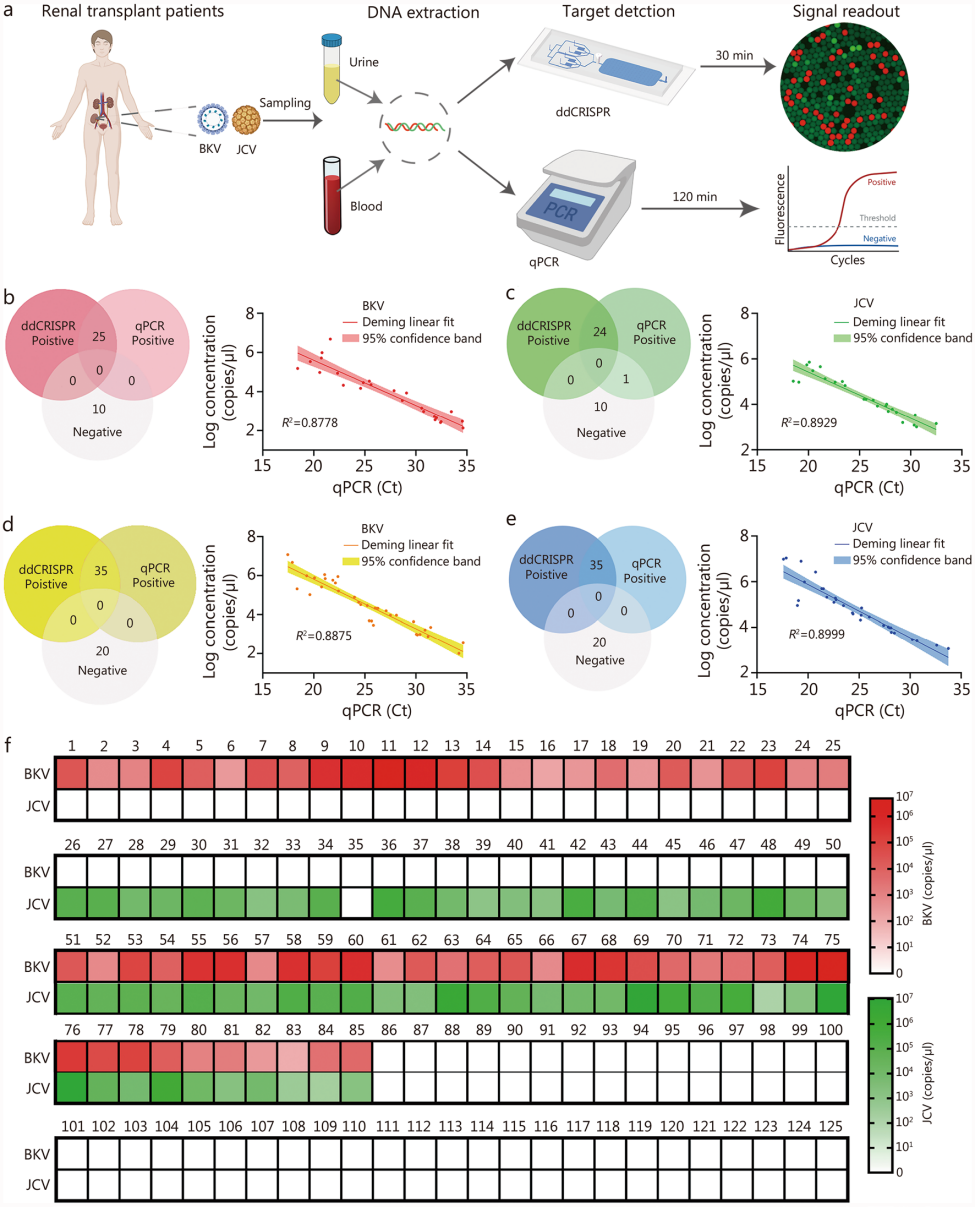
The clinical application of ddCRISPR. **a** Workflow for comparative analysis of BKV and JCV from blood and urine samples, using the ddCRISPR assay and qPCR. **b** Consistency (left) and correlation (right) between the chip assay and qPCR for BKV detection. **c** Consistency (left) and correlation (right) between the ddCRISPR assay and qPCR for JCV detection. **d** Consistency (left) and correlative (right) analysis of two methods for detecting BKV in co-infection cases. **e** Consistency (left) and correlative (right) analysis of two methods for detecting JCV in co-infection cases. **f** Heatmap showing the ddCRISPR results of cases 1 – 125. BKV BK virus, JCV JC virus, qPCR quantitative polymerase chain reaction, Ct cycle threshold, ddCRISPR dual-droplet microchip integrated with RPA-CRISPR

Subsequently, a total of 125 clinical samples were analyzed using ddCRISPR to investigate the potential application value of this assay in clinical practice. The experimental procedure was depicted in Fig. 4a, blood and urine samples from patients were collected for DNA extraction, and subsequent parallel detections were conducted using the ddCRISPR assay and the qPCR assay, respectively. The ddCRISPR assay was evaluated for the measurement of qPCR single positive samples from post-renal transplant patients with BKV or JCV infection (25 for each) and healthy controls (10 for each). Compared with qPCR, the sensitivity and specificity of the assay in detecting BKV were both 100% [Fig. 4b (left)]. For JCV detection, the assay showed high diagnostic performance with 96.0% (95% CI 80.5 − 99.3%) sensitivity and 100.0% (95% CI 74.1 − 100.0%) specificity [Fig. 4c (left)]. Moreover, a significant correlation was noted between the two approaches for detecting BKV and JCV, with determination coefficients *R*^*2*^ of 0.8778 [Fig. 4b (right)] and 0.8929 [Fig. 4c (right)], respectively.

Based on the above results, we proceeded to assess the simultaneous detection capability by measuring double-positive samples from post-renal transplant patients with BKV and JCV co-infection (*n* = 35) and healthy controls (*n* = 20). In terms of co-infection sample detection, the ddCRISPR assay maintained great performance [100.0% (95% CI 90.1 − 100.0%) sensitivity and 100.0% (95% CI 83.2 − 100.0%) specificity for both; Fig. 4d, e] and had a high correlation with qPCR in detecting BKV [*R*^*2*^ = 0.8875; Fig. 4d (right)] and JCV [*R*^*2*^ = 0.8999; Fig. 4e (right)]. The results for all samples were presented in Fig. 4f, showing high consistency between the results of the two methods. The only discrepancy was observed in sample No. 35, which was positive by qPCR but negative by the ddCRISPR assay. In this case, false-negative results may be due to the presence of interfering substances in the sample.

**Fig. 5:**
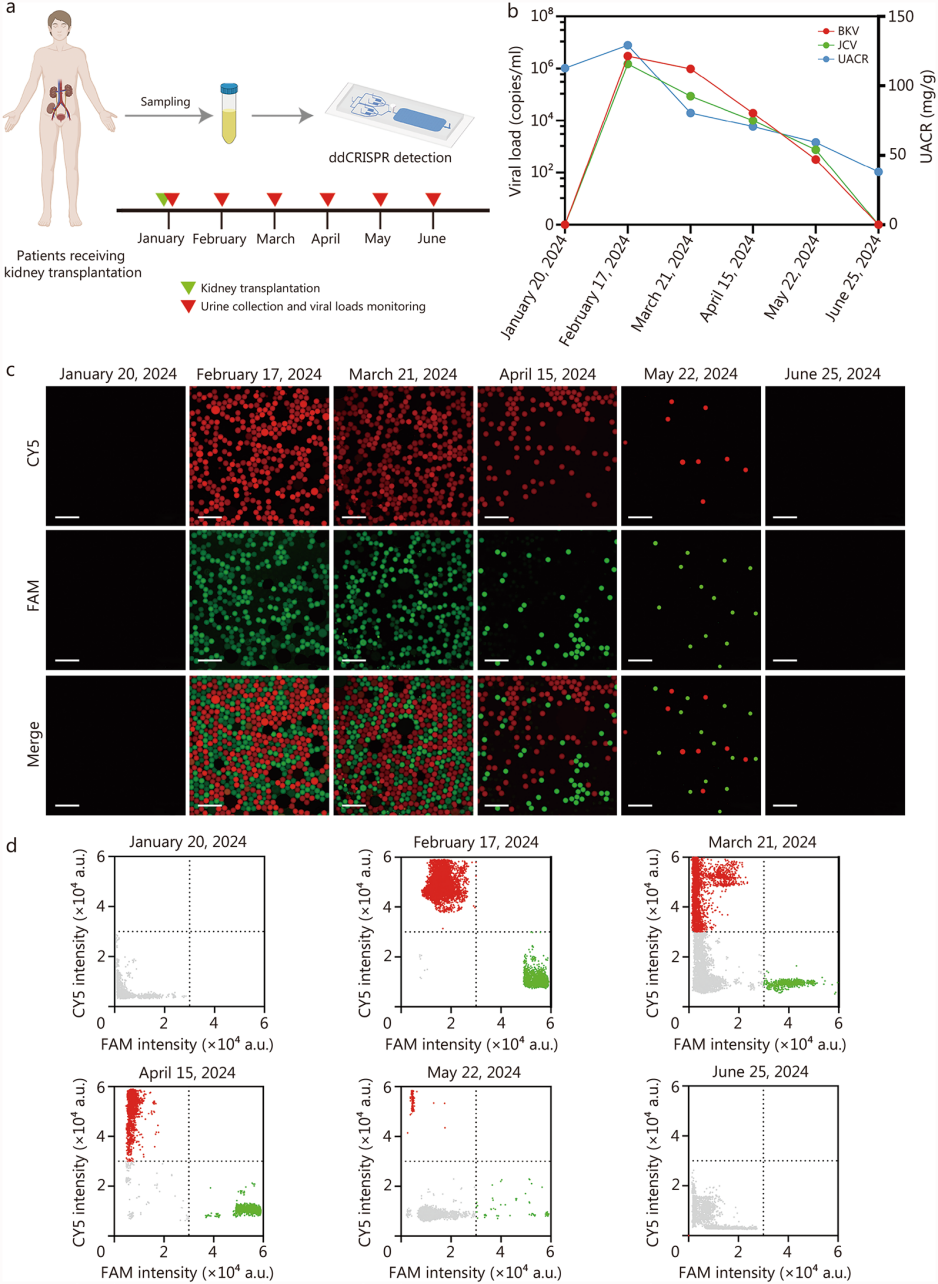
Viral load monitoring in a patient after kidney transplantation using ddCRISPR. **a** Schematic illustration of continuous viral load monitoring following kidney transplantation. Scale bar = 500 μm. **b** Monitoring of BKV and JCV infection and urine albumin-creatinine ratio (UACR) detection within 6 months after surgery. **c** Droplet results of this case monitored by ddCRISPR for 6 months. **d** Fluorescence scatter plots of this case monitored by ddCRISPR for 6 months. BKV BK virus, JCV JC virus, FAM 6-carboxyfluorescein, CY5 cyanine5, ddCRISPR dual-droplet microchip integrated with RPA-CRISPR

Among the medical management of post-kidney transplantation, it is significant to balance the immunosuppression stage of the recipient to prevent organ rejection and minimize the risk of transplantation-associated virus infection [34]. Thus, regular infection monitoring is essential for subsequent treatment plan adjustment. Herein, we investigated a patient co-infected with BKV and JCV, who presented with mild renal dysfunction following kidney transplantation, and monitored the viral load of BKV and JCV in the patient’s urine for 6 months using ddCRISPR (Fig. 5a). The patient presented with high levels of BKV and JCV in the early stage of infection. After adjusting the immunosuppressive drugs, the viral load decreased. Currently, the urinary albumin-creatinine ratio (UACR) is employed as an indicator of early renal function impairment [35]. Thus, UACR was employed in this study to evaluate kidney function, monitor the health status of the transplanted kidney, and analyze its association with viral load. As shown in Fig. 5b, the patient underwent kidney transplantation on January 20, 2024, and was administered immunosuppressive agents. High levels of BKV and JCV viral infection were identified in the patient on February 17, 2024, with a UACR of 112.8 mg/g. After adjusting the levels of immunosuppressants, the viral load and UACR gradually declined, and on June 25, 2024, BKV and JCV turned negative, and UACR was reduced to 38.0 mg/g. The droplet results and fluorescence scatter plots of this case monitored by ddCRISPR for 6 months are presented in Fig. 5c and d, respectively. The results indicate that the monitoring of BKV and JCV can be used for the efficacy observation and individualized, precise treatment of patients after kidney transplantation.

### Establishment and clinical application of the LFCRISPR assay

**Fig. 6:**
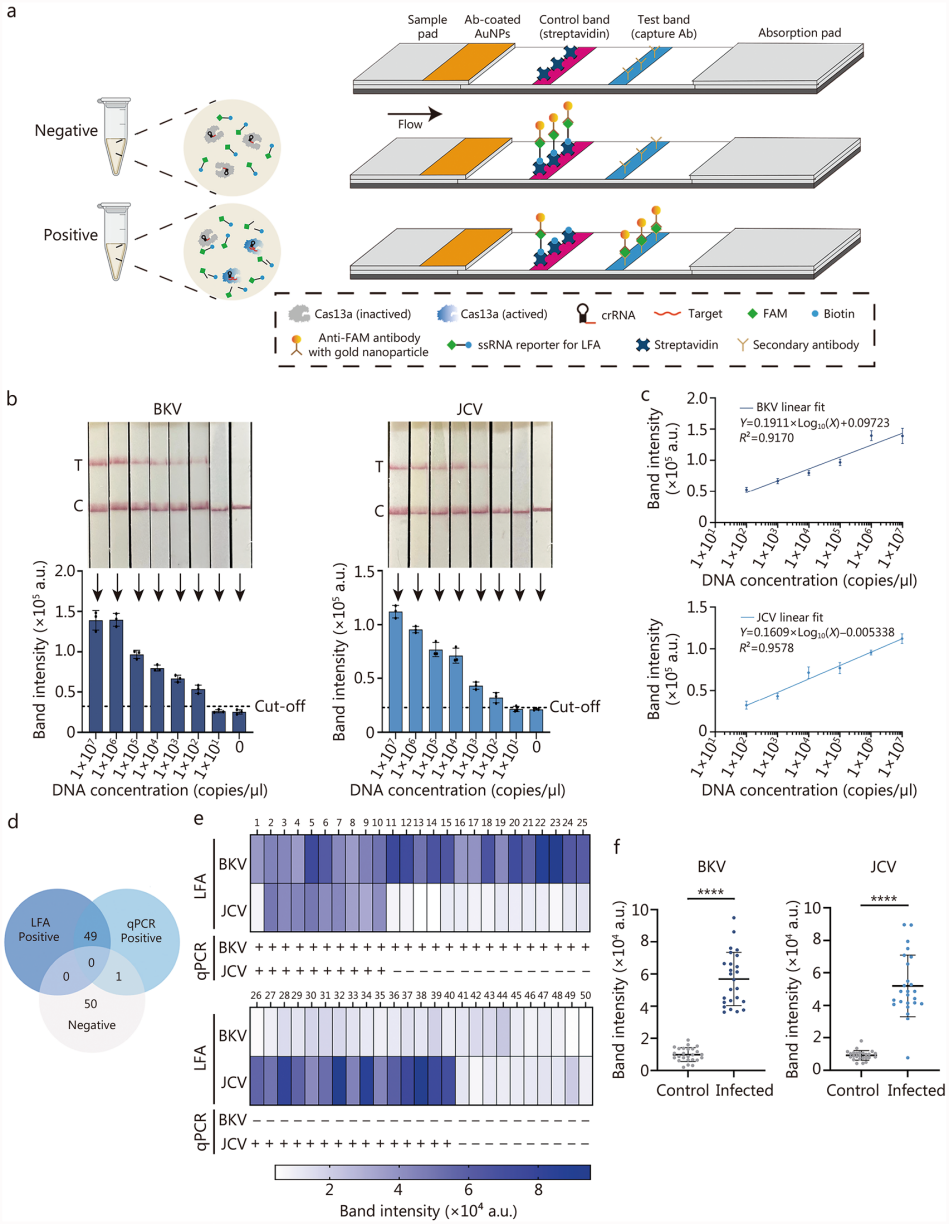
Establishment and clinical application of the LFCRISPR assay. **a** Schematic illustration depicts the principles of the LFCRISPR assay. In a positive test, it cleaves the FAM-biotin paired reporter and releases the FAM molecule for detection on the test band when Cas13a is activated by the target RNA. For a negative test, uncleaved reporters are captured through the binding of biotin to streptavidin on the control band. **b** The sensitivity of LFCRISPR with BKV and JCV standards (*n* = 3). **c** Calibrated standard curves for detection of BKV and JCV by RPA-CRISPR-LFA assay (*n* = 3). **d** Consistency between LFA and qPCR for analysis of clinical samples. **e** Heatmap showing the quantitative results for detecting BKV and JCV (corresponds to **d**). **f** Scatter plots for detecting BKV and JCV in the same clinical samples from healthy individuals and kidney transplantation recipients, as shown in **d**. *P*-values are calculated by a two-tailed Student’s *t*-test. ^****^P < 0.0001. Data are expressed as mean ± standard deviation (**b, c**). LFCRISPR lateral flow strip integrated with RPA-CRISPR, AuNPs gold nanoparticles, crRNA clustered regularly interspaced short palindromic repeats RNA, FAM 6-carboxyfluorescein, LFA lateral flow assay, BKV BK virus, JCV JC virus, qPCR quantitative polymerase chain reaction

Next, we combined CRISPR with lateral flow strips to create a fast, instant method for detecting BKV and JCV, which was called LFCRISPR. As illustrated in Fig. 6a, the ssRNA reporter is modified with a biotin and a FAM molecule at each end to enable its capture by gold nanoparticle-modified anti-FAM antibodies pre-loaded on the LFA strips. In the case of a negative test, complete reporters are captured upstream by streptavidin and subsequently labeled with gold nanoparticle-modified antibodies on the control band. And in the case of a positive test, cleaved reporters are capable of flowing downstream and are captured by the secondary antibodies specific to FAM to generate a positive signal on the test band. We assessed the sensitivity of LFCRISPR with BKV and JCV standards. As shown in Fig. 6b, the sensitivity of LFCRISPR for detecting BKV or JCV was 1 × 10^2^ copies/μl. Additionally, the findings indicated a significant correlation between the standard concentration and the band signal intensities of BKV (*R*^*2*^ = 0.9170) and JCV (*R*^*2*^ = 0.9578) detected by LFCRISPR (Fig. 6c), which meant that the method can only achieve semi-quantitative analysis by scanning with gray value or naked eye qualitative detection in clinical applications.

Subsequently, the clinical application value of LFCRISPR was verified by testing samples from patients confirmed by qPCR (10 co-infected with BKV and JCV, 15 positives for BKV, and 15 positives for JCV) and 10 healthy controls. The detection results of LFCRISPR are presented in Additional file 1: Fig. S7. A comparison of the overall results with qPCR is depicted in Fig. 6d, e, demonstrating the high consistency between these two methods. Notably, JCV was not detected by LFCRISPR in sample 1, and this method has restrictions for samples with Ct values exceeding 37 as detected by qPCR. Next, the greyscale values of the bands in the positive and negative samples were compared. As shown in Fig. 6f, the intensity of the positive band increased significantly in contrast to that of the negative band. The above results suggest that LFCRISPR is simple and easy to operate. It was found that the results can be interpreted by the naked eye without any equipment and can be utilized for the screening of BKV and JCV.

## Discussion

Among the various molecular diagnostic technologies for target sensing, isothermal amplification methods have been widely employed due to the virtue of simplicity, sensitivity, and robustness. Techniques such as RPA [36, 37], rolling-cycle amplification [38, 39], loop-mediated isothermal amplification [40, 41], and hybridization chain reaction [42] are also prominent in this field. In comparison to other methods, RPA is receiving increasing attention due to its low cost and mild reaction conditions. However, it is prone to result in non-specific amplification. To address this issue, the CRISPR-Cas system was introduced as a complementary tool. Overall, integrating the RPA-CRISPR reaction can consistently achieve detection performance comparable to that of qPCR without requiring complex operations and instruments. However, previous studies based on RPA-CRISPR assay have mostly achieved semi-quantitative detection by standard curve calibration [43–45], thus limiting precise quantification of significant targets and making it unfavorable for subsequent clinical decisions.

Opportunistic infection is the leading cause of graft loss, posing a great threat to allograft recipients. For renal transplant recipients, BKV and JCV represent the main opportunistic infection pathogens that can result in graft kidney injury or loss when rapid proliferation occurs in individuals with compromised immunity. Therefore, it is essential to monitor viral loads in renal transplant recipients promptly.

At present, qPCR is a widely adopted molecular diagnostic technique in clinical settings [46]. However, its application is limited by its relative quantification and moderate sensitivity, thereby impeding its efficacy in the precise detection of specific diseases or targets [47]. Considering the defects of current diagnostics, there is a pressing need for the development of non-invasive, personalized, and accurate diagnostic methods for the effective monitoring of organ transplant recipients. Thus, we reported a digital quantitative platform based on RPA/CRISPR-Cas13a, named ddCRISPR. As an innovative molecular diagnostic method, the whole reaction is driven only by a simple heating plate, which greatly reduces the instrument, detection costs, and the need for special laboratories while improving the detection efficiency, providing an alternative to qPCR for nucleic acid detection. By integrating the RPA/CRISPR-Cas13a assay into cell-sized droplets, ddCRISPR achieved simultaneous absolute quantification of dual targets without requiring a standard curve, while the compartmentalization effect within droplets enhanced local target concentration and amplified detection signals [48, 49]. Based on a previous study, compared to Cas12a, Cas13a could be easily integrated into the one-pot reaction, avoiding the pipetting step of the RPA/CRISPR-Cas12a two-step reaction, thus making it more compatible with subsequent digital droplet analysis and improving quantitative accuracy [50]. Additionally, it could achieve better detection outcomes than amplification-free diagnostics through the pre-amplification step [49, 51]. Our laboratory previously developed a microfluidic CRISPR/RPA droplet platform demonstrating high sensitivity for JCV detection [31]. Nevertheless, this method requires manual transfer of generated droplets to test tubes for CRISPR processing before subsequent detection on a secondary chip, a workflow susceptible to aerosol contamination through repetitive pipetting. Crucially, the system lacks multiplexing capability for the clinical differentiation of BKV and JCV co-infections. To overcome these technical constraints, we engineered an integrated microfluidic device enabling concurrent dual-target droplet generation and CRISPR-mediated detection. This innovation led to the development of ddCRISPR, a platform permitting simultaneous BKV/JCV quantification. The closed-system architecture eliminates manual transfers, thereby eradicating aerosolization risks while reducing processing steps. This method also achieves simultaneous quantitative detection of BKV and JCV, reducing detection time and cost. It not only assists physicians in better evaluating patients’ immune status and monitoring viral infections but also enhances detection efficiency and accuracy, ultimately providing improved medical services for patients. Although the RPA-CRISPR detection methods for BKV and JCV have been optimised, the higher sensitivity observed in JCV detection may partly be due to the use of FAM as a fluorescent dye. Compared to CY5, which is used for BKV detection, FAM exhibits a higher fluorescence quantum yield and detection efficiency. This explains why ddCRISPR detection is more sensitive for JCV than for BKV. This issue also needs to be addressed in subsequent work.

By combining an RPA-CRISPR assay with commercially available test strips, we developed an RPA-CRISPR-LFA-based assay, called LFCRISPR. This approach maintained the detection performance of CRISPR-based diagnostics while simplifying the operation procedures. Furthermore, considering the isothermal reaction of the RPA-CRISPR assay, it is easily adaptable to be developed as a point-of-care test suitable for resource-limited settings. The sensitivity of the LFCRISPR assay is 100 copies/μl, which, although not exceptionally high, demonstrates significant clinical value for BKV and JCV detection in the context of organ transplantation. This method enables rapid screening for BKV and JCV infections post-transplantation, thereby assisting clinicians in timely adjusting treatment strategies. Moreover, healthcare professionals can implement this technology in primary care settings or during postoperative follow-up to facilitate early patient intervention, ultimately reducing the risk of complications. Additionally, the LFCRISPR assay is characterized by its low cost, making it suitable for large-scale screening efforts while enhancing overall diagnostic efficiency. As a versatile platform, ddCRISPR can facilitate the multiplex detection of various pathogens and tumor biomarkers by establishing target-specific detection systems. This capability holds significant potential for molecular diagnostics in infectious diseases and cancer. However, LFCRISPR could only achieve single detection due to the limitations of detecting antibodies on test strips. LFA has been successfully established for dual-target detection [52], our work will focus on developing an advanced LFCRISPR system for simultaneous multiplex target detection.

## Conclusions

In brief, our study presents two distinct detection platforms for BKV and JCV detection in post-kidney transplant recipients and PVAN patients. Thus, doctors can promptly and comprehensively understand the infection and immune status of patients, improve diagnostic efficiency, and take diagnostic and treatment measures in a timely manner. We anticipate that these two RPA-CRISPR-based technologies, with further refinement, will hold promise as versatile tools in clinical practice and universal detection platforms for diverse nucleic acid biomarkers.

## Supplementary Information

**Additional file1. Methods. Table S1** Sequence of RPA primers, crRNAs, and ssRNA reporters. **Fig. S1** Optimization of LwaCas13a concentration. **Fig. S2** Sequencing results of RPA products. **Fig. S3** Heatmap of fluorescent results between matched and mismatched crRNA and plasmid for specificity evaluation. **Fig. S4** Evaluation of droplet generation capacity and reproducibility of the microchip. **Fig. S5** qPCR sensitivity of BKV and JCV detection using a commercial kit. **Fig. S6** Representative images of ddCRISPR specificity evaluation using different viruses. **Fig. S7** Clinical samples detection using LFCRISPR.

**Additional file2. Video S1** Droplet formation in the generation unit of a droplet microfluidic chip

**Additional file3. Video S2** Droplet collection in the detection zone of a droplet microfluidic chip

## Data Availability

The data and materials used in the current study are all available from the corresponding author upon reasonable request.

## Abbreviations

BKV: BK virus
CRISPR: Clustered regularly interspaced short palindromic repeats
CMV: Cytomegalovirus
crRNA: Clustered regularly interspaced short palindromic repeats RNA
CY5: Cyanine5
dsDNA: Double-stranded DNA
ddCRISPR: Dual-droplet microchip integrated with RPA-CRISPR
EBV: Epstein-Barr virus
FAM: 6-Carboxyfluorescein
HPyV6: Human polyomavirus 6
HPyV7: Human polyomavirus 7
JCV: JC virus
KIPyV: Karolinska Institute polyomavirus
LFA: Lateral flow assay
LFCRISPR: Lateral flow strip integrated with RPA-CRISPR
MCPyV: Merkel cell polyomavirus
NTP: Nucleoside triphosphate
PyV: Polyomaviruse
PDMS: Poly(dimethylsiloxane)
PML: Progressive multifocal leukoencephalopathy
PVAN: Polyomavirus-associated nephropathy
PyVs: Polyomaviruses
qPCR: Quantitative polymerase chain reaction
RPA: Recombinase polymerase amplification
ssRNA: Single-strand RNA
UACR: Urinary albumin-creatinine ratio
WUPyV: Washington University polyomavirus
