# Supplementary material for: CRISPR-based assays for the detection of BK virus and JC virus infections post-kidney transplantation
Source: Mil Med Res. 2025 Aug 4;12:44. doi: 10.1186/s40779-025-00632-0 (PMC12320373; doi:10.1186/s40779-025-00632-0)
Supplement: Supplementary file 1 — Additional file1. Methods. Table S1 Sequence of RPA primers, crRNAs, and ssRNA reporters. Fig. S1 Optimization of LwaCas13a concentration. Fig. S2 Sequencing results of RPA products. Fig. S3 Heatmap of fluorescent results between matched and mismatched crRNA and plasmid for specificity evaluation. Fig. S4 Evaluation of droplet generation capacity and reproducibility of the microchip. Fig. S5 qPCR sensitivity of BKV and JCV detection using a commercial kit. Fig. S6 Representative images of ddCRISPR specificity evaluation using different viruses. Fig. S7 Clinical samples detection using LFCRISPR. [file 40779_2025_632_MOESM1_ESM.pdf]

Methods

Sample collection

Blood or urine samples were gathered from 85 kidney transplant recipients and 40 healthy individuals at Renji Hospital from January 2024 to May 2025. BKV and JCV were identified by qPCR and ddCRISPR assay in all the samples. All samples used in this study followed the informed consent and agreement of patients as well as the ethical regulations of Shanghai Jiaotong University School of Medicine Renji Hospital (LY2025-224-B). The clinical study was registered at the Chinese Clinical Trial Registry (Registration No. ChiCTR2500106093) during the preliminary phase.

Pretreatment of clinical samples

All clinical samples were prepared according to the manufacturer's instructions for the viral nucleic acid quantitative detection kit. For blood or plasma, 100 μl samples and 50 μl concentrated solution were mixed and centrifuged (13,000 rpm, 10 min). And the sediment was treated with 25 μl lysis solution. For urine, 1 ml samples were centrifuged first (13,000 rpm, 10 min). After removing the supernatant, the sediment was treated with 50 μl lysis solution. After the steps above were finished, the mixture of sediment and lysis solution was heated at 100 °C for 10 min. Finally, the supernatant containing purified nucleic acid was obtained by centrifugation (13,000 rpm, 10 min). The prepared samples were stored at –20 °C before use.

RPA assay

For the RPA assay, the RPA amplification kit was used according to the manufacturer's instructions. The reaction mix was prepared, including 25 μl RPA buffer, 14 μl ddH_2O, 2 μl forward primer (10 μmol/L), 2 μl reverse primer (10 μmol/L), and 4 μl DNA template. Then, the reaction mix was transferred to an RPA freeze-dried powder tube to dissolve it fully. Finally, after adding 3 μl initiator containing Mg^{2+}, the RPA reaction was initiated and performed at 37 °C for 20 min. And the amplicons were ready for subsequent Cas13a assay or electrophoretic analysis.

Cas13a assay

Detection of RPA amplified products was performed by Cas13a cleavage detection according to the manufacturer's instructions with minor modifications. The total reaction volume was 50 μl, including 5 μl 10× cleavage buffer, 5 μl NTP (10 μmol/L), 4 μl ssRNA reporter, 2 μl crRNA with different final concentrations, 2 μl Cas13a with different concentrations, 1 μl RNase inhibitor (30 U/μl), T7

transcriptase (40 U/μl), 4 μl RPA products, and 24.4 μl ddH_2O. In this procedure, we set 5, 10, 20, 40, 80, and 200 nmol/L as final concentrations of crRNA and 20, 40, 80, 100, 200, and 400 nmol/L as final concentrations of Cas13a to optimize the reaction system. The fluorescence was measured by a real-time PCR system (ABI QuantStudio5, Thermo Fisher Scientific Inc., Waltham, MA, USA) or microplate reader (Synergy H1, BioTek Instruments, Inc., Winooski, VT, USA) for 30 min at 37 °C.

qPCR assay

The PCR premix was prepared, which contained 8.5 μl PCR mix, 0.5 μl enzyme mix, 10 μl reinforcing agent, and 1 μl internal standard for each reaction. Then, 5 μl of the prepared samples were added to the premix. The reaction program consisted of 95 °C for 3 min, 40 cycles of 94 °C for 15 s and 60 °C for 35 s.

Table S1 Sequence of RPA primers, crRNAs, and ssRNA reporters

**Name**	**Sequence (5′ – 3′)**
BKV-F1	CATGGAATGCAGCCAAACAGGAAGGAAAGTG
BKV-F2	CAGCCAGCCAGTGGCAGTTAATAGTGAAACC
BKV-F3	CCTCAGAAAAAGCCTCCACACCCTTACTAC
BKV-R1	CTTGTCGTGACAGCTGGCGTAGAACCATGGC
BKV-R2	CAAGGCCAAGATTCCTCGGCTCGCAAAAC
BKV-R3	CTATTAACTGCCACTGGCTGGCTGCCCAGTC
JCV-F1	CAATTTCTATATCAGATACATTTGAAAGTGAC
JCV-F2	CAAATAAGGACATGCTTCCTTGTTACAGTG
JCV-F3	CATTTGAAAGTGACTCCCCAAATAAGGACATG
JCV-R1	CATTGTCATGAGCTGCTTGACCATTGGAGTGC
JCV-R2	CTTGACCATTGGAGTGCACATTCATCAAAGT
JCV-R3	CATTGTCATGAGCTGCTTGACCATTGGAGTG
BKV-crRNA1	GAUUUAGACUACCCCAAAAACGAAGGGGACUAAAACCACA AGAGGAAGUGGAAAGUAGCCAAAG
BKV-crRNA2	GAUUUAGACUACCCCAAAAACGAAGGGGACUAAAACUGU GUUUAUUUGAGAAUUCUAGGGGCG
BKV-crRNA3	GAUUUAGACUACCCCAAAAACGAAGGGGACUAAAACCACU UCCUCUUGUGUUUAUUUGAGAAU
JCV-crRNA1	GAUUUAGACUACCCCAAAAACGAAGGGGACUAAAACCAAU CUAAAUGAGGAUCUAACCUGUGGA
JCV-crRNA2	GAUUUAGACUACCCCAAAAACGAAGGGGACUAAAACGAA UUCUGGCCACACUGUAACAAGGAA
JCV-crRNA3	GAUUUAGACUACCCCAAAAACGAAGGGGACUAAAACUUU AGAUUGGGUAGUGGAAUUCUGGCC
T7 promoter	TAATACGACTCACTATAGGG
ssRNA reporter for fluorescence readout	FAM-UUUUU-BHQ1<br>CY5-UUUUU-BHQ2
ssRNA reporter for LFA	FAM-UUUUUUUUUUUU-Biotin

RPA recombinase polymerase amplification, *crRNA* clustered regularly interspaced short palindromic

repeats RNA, *ssRNA* single-strand RNA, *BKV* BK virus, *JCV* JC virus, *F* forward primer, *R* reverse primer, *LFA* lateral flow assay

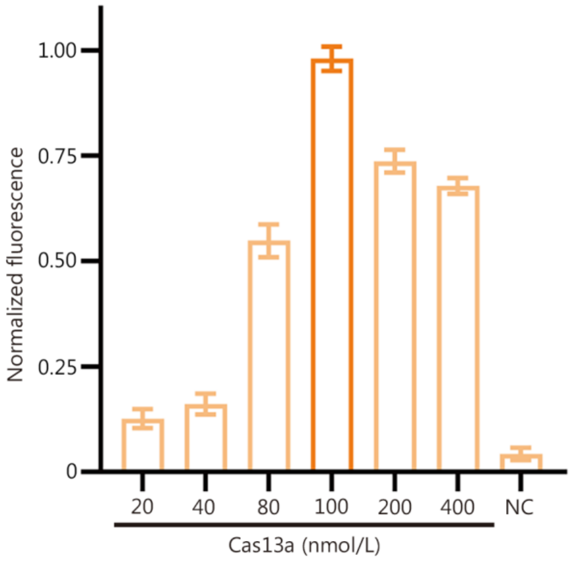

Fig. S1 Optimization of LwaCas13a concentration. NC negative control

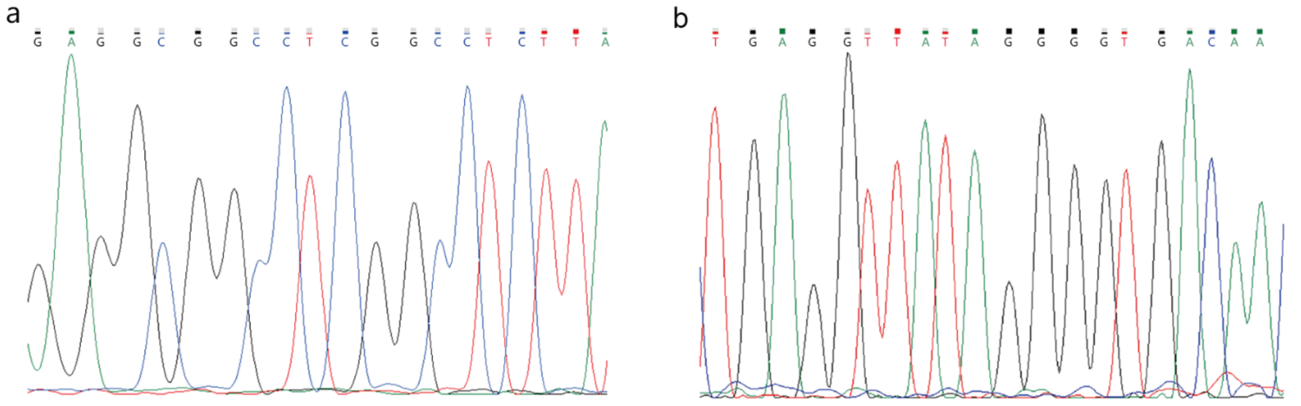

Fig. S2 Sequencing results of RPA products. **a** BKV RPA product using primer F2R1. **b** JCV RPA product using primer F3R2. RPA recombinase polymerase amplification, BKV BK virus, JCV JC virus

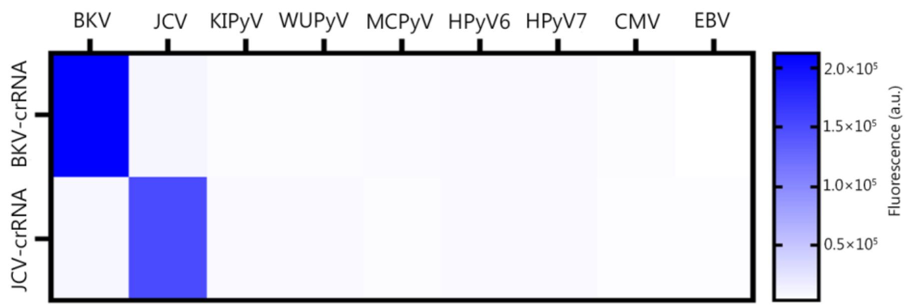

Fig. S3 Heatmap of fluorescent results between matched and mismatched crRNA and plasmid for specificity evaluation. crRNA clustered regularly interspaced short palindromic repeats RNA, BKV BK virus, JCV JC virus, KIPyV Karolinska Institute polyomavirus, WUPyV Washington University polyomavirus, MCPyV Merkel cell polyomavirus, HPyV6 human polyomavirus 6, HPyV7 human polyomavirus 7, CMV cytomegalovirus, EBV Epstein-Barr virus

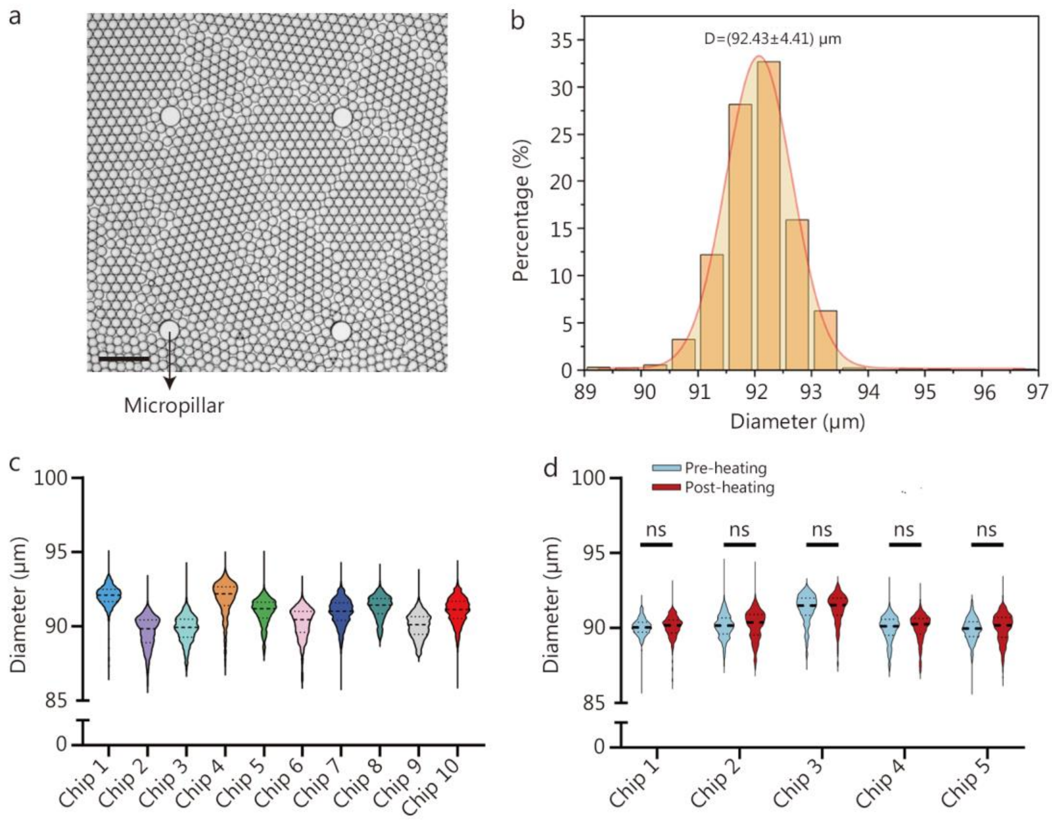

Fig. S4 Evaluation of droplet generation capacity and reproducibility of the microchip. **a** Microscopic photograph of picoliter-sized droplets. Scale bar = 500 μm. **b** Size distribution of the droplets. **c** Reproducibility assessment of droplet size generated per experiment. **d** Effect of temperature factor on droplet size. D diameter, ns non-significance

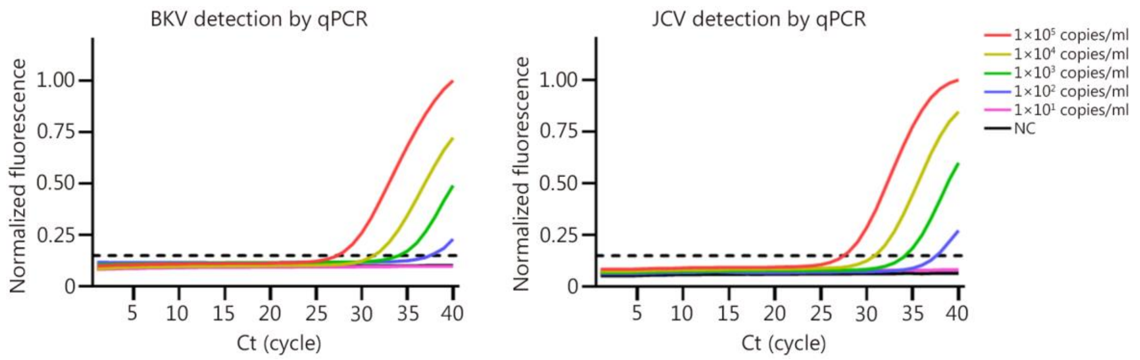

Fig. S5 qPCR sensitivity of BKV and JCV detection using a commercial kit. BKV BK virus, JCV JC virus, qPCR quantitative polymerase chain reaction, NC negative control

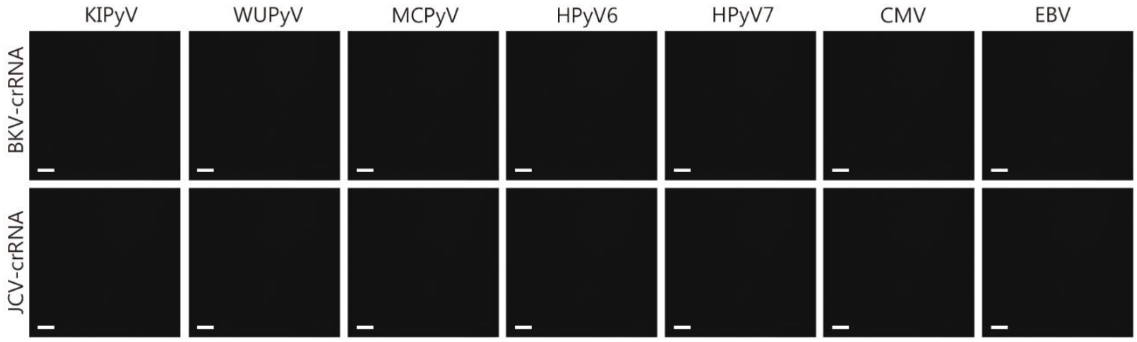

Fig. S6 Representative images of ddCRISPR specificity evaluation using different viruses. Scale bar = 400 μm. BKV BK virus, JCV JC virus, KIPyV Karolinska Institute polyomavirus, WUPyV Washington University polyomavirus, MCPyV Merkel cell polyomavirus, HPyV6 human polyomavirus 6, HPyV7 human polyomavirus 7, CMV cytomegalovirus, EBV Epstein-Barr virus

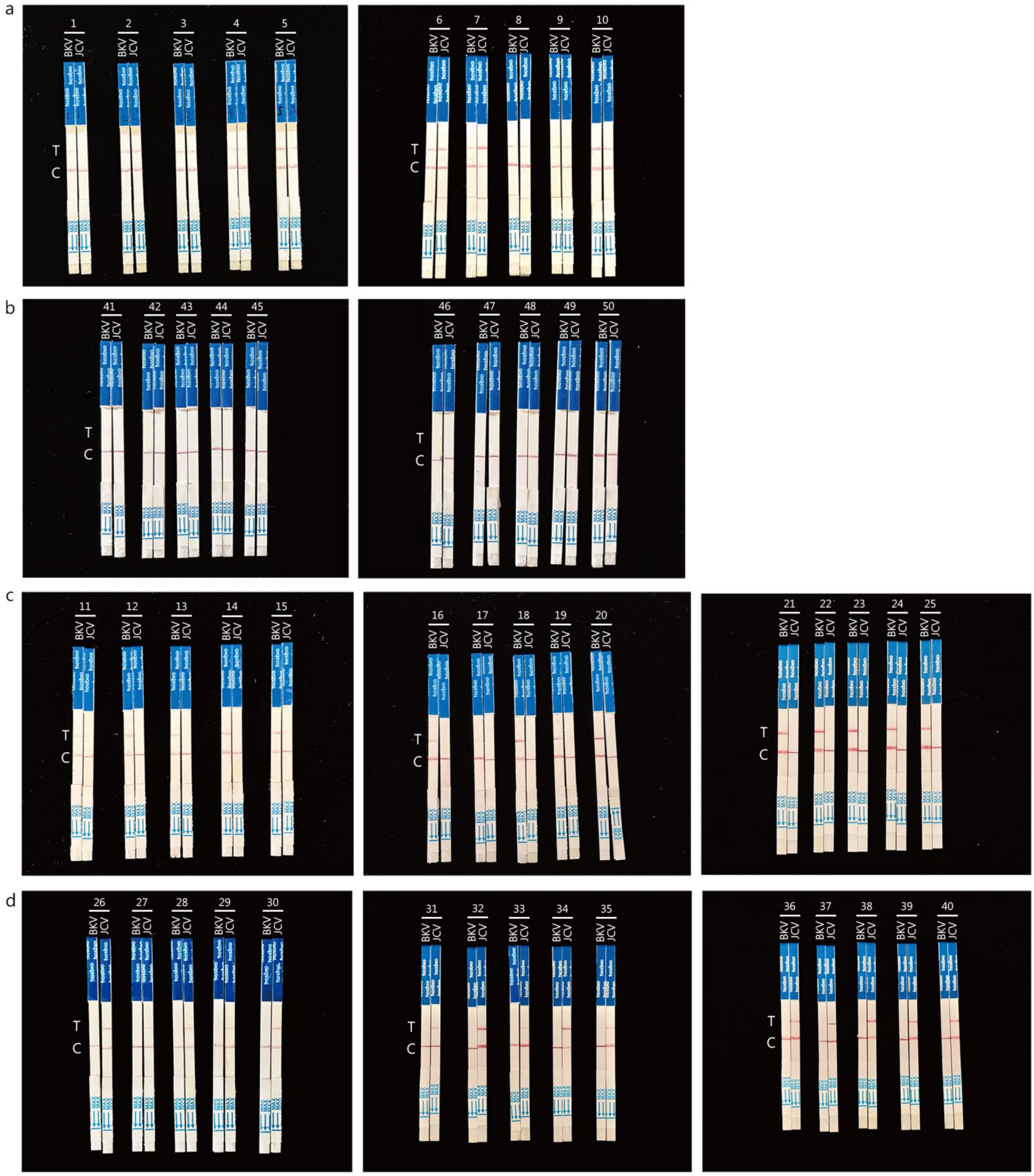

Fig. S7 Clinical samples detection using LFCRISPR. **a** The results of double-positive samples. **b** The results of double-negative samples. **c** The results of BKV-positive samples. **d** The results of JCV-positive samples. LFCRISPR lateral flow strip integrated with RPA-CRISPR, BKV BK virus, JCV JC virus
